# New onset asthma during pregnancy: two case reports

**DOI:** 10.12688/f1000research.73188.1

**Published:** 2021-11-08

**Authors:** Paula S. Schriek, Saar A. Bendien, Hanneke A. Feitsma, Jeroen van Exsel

**Affiliations:** 1 Department of Respiratory Medicine, Haga Teaching Hospital, The Hague, South Holland, 2545AA, The Netherlands; 2 Department of gynaecology, Haga Teaching Hospital, The Hague, South Holland, 2545AA, The Netherlands

**Keywords:** asthma, pregnancy, infection, exacerbation

## Abstract

Introduction:

Asthma is the most common chronic respiratory disease during pregnancy. However, reports of new onset asthma during pregnancy are lacking. We report two cases of new onset asthma during pregnancy following respiratory tract infection, subsequently one case with M. pneumoniae infection and the other case with a combined infection with respiratory syncytial virus and rhinovirus.

Case presentation:

Both patients presented with the clinical features of an acute asthma exacerbation during pregnancy without a medical history of asthma. During follow up the diagnosis of asthma was supported by spirometry showing significant reversibility and elevated fractional exhaled nitric oxide (FeNO). Patients were hospitalized and received supplemental oxygen, treatment for an acute asthma exacerbation with systemic corticosteroids, high dose inhalation therapy. These therapeutic interventions subsequently led to a good outcome for the mother and newborn in both cases.

Conclusions:

New onset asthma should be part of the differential diagnosis in pregnant patients with respiratory symptoms, particularly in case of mycoplasma infection.

Diagnosing asthma during pregnancy can be challenging. In these circumstances, additional diagnostic tests (like inflammatory biomarkers FeNO and blood eosinophils) can be helpful to support the diagnosis.

## Abbreviations

b.i.d., twice a day; body mass index, BMI; coronavirus disease 2019, COVID-19; C-Reactive Protein test, CRP; Estimated fetal weight, EFW; FEV1, forced expiratory volume in 1 s; FVC, forced vital capacity; FENO, fractional exhaled nitric oxide; gestational age, GA; immunoglobulin M, IgM; inhaled corticosteroid, ICS; OCS, oral corticosteroids; respiratory syncytial virus, RSV; severe acute respiratory syndrome coronavirus, SARS-CoV-2

## Introduction

Asthma is one of the most common chronic diseases during pregnancy with a prevalence of 7–10%. Dyspnoea is a frequent symptom, being present in 60–70% of healthy pregnancies, mainly as a result of normal physiological changes ( Table 1) ^1^. When a patient presents with dyspnoea during pregnancy it can be challenging to distinguish dyspnoea related to physiological changes (such as decrease in functional residual capacity and pregnancy-induced hyperventilation ( Table 1.)) from pathology, such as uncontrolled asthma. This can be particularly difficult in patients without a medical history of asthma. Reports of new onset asthma during pregnancy are lacking. Uncontrolled asthma and asthma exacerbations during pregnancy are associated with increased risk of adverse outcomes for mother and child such as preterm birth, low birth weight and pre-eclampsia ^2, 3^. Viral respiratory infections are the most common trigger for asthma exacerbations. *M. pneumoniae* infection is also known to play a role in asthma, resulting in exacerbations, and possibly related to the pathogenesis of asthma ^4, 5^. It is thought that pregnant women are more susceptible to infections or that they are more severely affected by infectious diseases ^6, 7^. In this report, we describe two patients with *de novo* diagnosis of asthma during pregnancy following respiratory tract infection.

**Table 1.** Differential diagnosis of dyspnoea during pregnancy.

**Pathological**
Pulmonary embolism
Pneumonia
(new onset) Asthma
Pre- eclampsia with pulmonary oedema
Peripartum cardiomyopathy
Dysfunctional breathing
Pneumothorax
Amniotic fluid embolism
**Physiological (changes during pregnancy)**
Pregnancy related hyperventilation
Decreased functional residual capacity (FRC)
Decreased expiratory reserve volume (ERV)
Anaemia

## Case reports

### Case one

A healthy 33-year-old Caucasian woman presented to the emergency department with progressive dyspnoea in her third trimester of pregnancy, at 32 weeks gestation. The general practitioner treated her with clarithromycin, salbutamol aerosol 100 μg/do and oral corticosteroids (OCS) (prednisone 30mg) and beclomethasone 100 μg/do metered-dose inhaler twice daily (b.i.d.). Despite this therapy, her clinical condition deteriorated with progressive work of breathing and cough.

Her medical history consisted of scoliosis and one episode of possible pneumonia, treated by her primary care physician with antibiotics. Independent of this event, she never experienced respiratory symptoms. She had no family history of asthma and no personal history of atopy. She stopped smoking 5 years previously. Her body mass index (BMI) was 24 mg/m ^2^. She was employed as a teacher. 

At presentation, the patient was in moderate respiratory distress. Vital signs showed an oxygen saturation of 98% on room air and blood pressure of 130/70mmHg. 

Lung auscultation revealed an expiratory wheeze with scattered rhonchi. The physical examination and chest radiograph showed no abnormalities. The white-blood-cell count was 16.3×10 ^9^/L, with neutrophils of 15.3×10 ^9^/L, and eosinophils of 0.05 ×10 ^9^/L. C-Reactive Protein test (CRP) was low (4 mg/L). Arterial blood gas analysis showed respiratory alkalosis with pH 7.46, pCO _2_ 3.6 kPa, HCO _3_^−^ 21 mmol/L, pO _2_ 11.6kPA, with an elevated A-a gradient of 3.9 kPa.

Sputum cultures showed upper respiratory tract flora. Serum immunoglobulin M (IgM) antibody titer was 16 for M *. pneumoniae*, which is indicative of current infection. Allergy testing with Phadiatop test was negative (Total serum IgE: 33 IU/ml).

The patient was admitted to the hospital due to respiratory distress and treated with prednisone 30mg/day for 10 days, clarithromycin was increased to 500 mg b.i.d. in combination with salbutamol/ipratropium 1/0.2 mg/ml inhalation solution every four hours. The beclomethasone inhaler was converted to a high dose inhaled corticosteroid and a long-acting beta-agonist (ICS/LABA) (beclomethasone/formoterol aerosol 200/6 µg, two inhalations b.i.d.). Throughout the course of the week, the patient remained tachypnoeic, oxygen dependent and frequent nebulizing was needed. After a week, her symptoms improved and OCS was systematically tapered. Daily cardiotocography and biweekly fetal growth ultrasound were normal, with an estimated fetal weight (EFW) of p57 and normal amniotic fluid. The patient was discharged after a hospital stay of 15 days.

## Follow-up and outcomes

Two weeks after discharge, and discontinuation of OCS, spirometry revealed FVC 3.37L (84%), FEV1 2.75L (82%) pre bronchodilator (pre-BD), FEV1 3.12L (93%) post bronchodilator (post-BD). Spirometry demonstrates significant reversibility (13% and 262 cc) which is compatible with asthma. Furthermore, strongly elevated fractional exhaled nitric oxide (FeNO) (60ppb) and elevated blood eosinophils levels (0.50×10 ^9^/L) were found. Due to persistent eosinophilic inflammation, treatment with ICS was intensified. FeNO normalized after 8 weeks (18ppb). The outcomes after pregnancy were remarkably good for the patient and newborn. No maternal complications during labor or late term pregnancy occurred. The patient gave birth to a healthy baby boy at a GA of 39 weeks and three days, with a birthweight of 3445 grams. The baby had an uncomplicated neonatal course. Over a period of 6 months, inhalation medication could be reduced to ICS only and the patient was referred back to primary care.

### Case two

A 22-year-old Turkish woman, with morbid obesity (BMI of 46) and no further medical history, presented to the emergency department with dyspnoea and a productive cough in her first trimester of pregnancy, at 8 weeks gestation. She developed a cold with nasal congestion, a productive cough and progressive dyspnoea. There was no family history of asthma and she had never smoked. She was employed as a housekeeper. 

At presentation, vital signs showed a respiratory rate of 23 breaths/minute, oxygen saturation of 92% on room air and blood pressure of 135/85. Lung auscultation revealed an extended expirium with scattered rhonchi. The chest radiograph showed no signs of pneumonia. The white-blood-cell count was 12.6×10 ^9^/L, with neutrophils of 10.0×10 ^9^/L and eosinophils of 0.28×10 ^9^/L. CRP was elevated; 76 mg/L. Arterial blood gas analysis showed hypoxemia and respiratory alkalosis with pH 7.44, pCO _2_ 4.0 kPa, HCO _3_ 22 mmol/L, pO _2_ 8.4 kPa, with an elevated A-a gradient of 6.6 kPa.

The patient was admitted to the hospital. Treatment was started with prednisone 30 mg/day, clarithromycin 500 mg b.i.d. in combination with salbutamol/ipratropium 1/0.2 mg/ml nebulizer and inhaled oxygen. High dose beclomethasone/formoterol aerosol 200/6 µg was started with two inhalations b.i.d. Polymerase chain reaction on throat swab was positive for respiratory syncytial virus (RSV) and rhinovirus. Phadiatop test was positive with increased IgE for dog rose, house dust mite, timothy grass and birch pollen (total serum IgE level 1632 /IU/ml).

Spirometry during hospitalization revealed FEV1 2.47L (78%) and FEV1/FVC (84.31%), Z-score of -0.68. The patient gradually recovered, and antibiotics, prednisone and salbutamol/ipratropium inhalation were discontinued after three days. The patient was discharged after six days.

One month after discharge she returned to our emergency department with complaints of a common cold with a sore throat, a productive cough and dyspnoea. At presentation, the patient was not clinically in distress and there was no hypoxemia. Treatment was started with prednisone 30 mg/day for seven days and the patient was discharged. Spirometry performed in the outpatient setting revealed FEV1 1.81L (57%) pre-BD, FEV1 2.07L (66%) post-BD, FEV1/FVC (70.29%, Z-score: -2.53). Spirometry demonstrates significant reversibility (15% and 260 cc) which supports the diagnosis of asthma. Furthermore, FeNO was strongly elevated (58ppb). At 20 weeks gestational age (GA), spirometry revealed FEV1 3.25L (103%) and FEV1/FVC (86.44%, Z-score: -0.32), demonstrating significant variability over time. 

The patient developed gestational diabetes mellitus in the third trimester. Monthly growth ultrasound showed normal fetal growth (EFW p72), normal abdominal circumference (fetal abdominal circumference p44) and normal amniotic fluid.

## Follow-up and outcomes

At 28 weeks, the patient presented to our emergency department again with dyspnoea and a non-productive cough. She was clinically stable with an adequate oxygen saturation of 97%. The blood eosinophil count was 0.36×10 ^9^/L. with a CRP of 23 mg/L. Newly diagnosed was an iron deficiency anaemia (haemoglobin: 5.6 mmol/L) which recovered after ferricarboxymaltose 1000mg (haemoglobin afterwards 7.6mmol/L). She was admitted to the hospital and treated with prednisone 30 mg/day for five days and salbutamol/ipratropium 1/0.2 mg/ml inhalation. Both sputum cultures and serum IgM for *M. pneumoniae* were 

During admission, daily fetal assessment was performed, ultrasound scan of fetal growth was normal. The patient was discharged after seven days. the pregnancy outcomes of the patient were normal. She gave (uncomplicated vaginal) birth to a healthy baby girl at a GA of 41+4 with a birthweight of 3805 grams. The apgar score was 9-10-10 after 1,5 and 10 minutes. 

In the outpatient setting the patient received two more follow-ups from the respiratory physician and the specialized respiratory nurse. During these consultations no more asthma exacerbations were reported but unfortunately the patient’s adherence to medication seemed poor. After these consultations the patient was lost to follow up in secondary care. Further monitoring of asthma will be performed by primary care. 

## Discussion

New onset asthma during pregnancy may not always be recognized but might be rather common. Based on pulmonary function testing, inflammatory biomarkers and clinical response to treatment, our patients are strongly suspected to have new onset asthma triggered by infection during pregnancy. To the best of our knowledge, there are no previous case reports about new onset asthma during pregnancy following mycoplasma infection. Both patients presented with severe exacerbations requiring hospitalization, oxygen supplementation and treatment with OCS and antibiotics. These interventions subsequently led to a good outcome for the mother and newborn in both cases. This illustrates the importance of active treatment of asthma exacerbations during pregnancy.

The diagnosis of asthma during pregnancy can be challenging as bronchoprovocation testing with metacholine or histamine is contra indicated during pregnancy ^8^. Healthy pregnant women show no change in FEV1 and FVC during (late) pregnancy. So, in patients suspected of new onset asthma during pregnancy, and normal spirometry, repeating spirometry when the patient is having more symptoms (as in case 2) to demonstrate significant variability or additional inflammatory biomarkers (as in case 1) can support the diagnosis. This is important since over- as well as under-treatment should be avoided in pregnant patients with asthma.

In non-pregnant women an exacerbation, which can be triggered by viral infections, may be the presenting manifestation of asthma. Mycoplasma infection resulting in new onset, infection- mediated, asthma, outside pregnancy, is also well recognized ^4^. These two phenomena are not as well described in pregnancy. Current literature suggests that *M. pneumoniae* is an important trigger for acute exacerbations of asthma, accounting for 3.3–50% of all exacerbations. However, the role of *M. pneumonia* infection in the pathogenesis of asthma is still controversial ^5, 9^. In 1994, Yano *et al.*, reported a case of mycoplasma infection leading to a first presentation of asthma, suggesting the infection led to a complex interplay of airway inflammation and IgE mediated hypersensitivity (type 1 reaction as seen in asthma) ^10^. Recent studies also show that *M. Pneumoniae* infection in non-pregnant women and children can contribute to the development of asthma and prevalence of asthma is increased in patients with a history of atypical pneumonia ^1, 4, 5, 11^. Although these findings are suggestive of an association between M. pneumoniae and asthma disease onset and exacerbations, larger well controlled studies are needed to test this hypothesis and identify underlying mechanisms.

The role of other viral respiratory tract infections, especially RSV or rhinovirus, in the development of asthma is also a subject of interest. Certain components seem to contribute to the risk of developing asthma, such as type and severity of viral infections and host characteristics ^12^. It is well known that pregnant women are more susceptible to viral infections and that they are more at risk for developing severe diseases. For pregnant women with asthma this risk is even higher ^13^

Pregnancy is a pro-inflammatory and immune modulating condition, depending on the stage of pregnancy ^6, 7, 14, 15^. Theoretical, physiological immunosuppression is a characteristic of pregnancy and results in feto-maternal tolerance, although this is a complex interacting network and not yet fully understood. Changes in immune characteristics during pregnancy including a shift in (Th1)/Th2 ratio toward a Th2- predominant immune state and an increase in regulatory T cells (Tregs) may explain increased susceptibility to infections ^16, 17^. These alterations in immune function possibly also have a role in triggering the process of developing new onset asthma during pregnancy.

The current severe acute respiratory syndrome coronavirus 2 (SARS-CoV-2) pandemic that caused the coronavirus disease 2019 (COVID-19), resulted in many deaths worldwide. Recent studies suggest that pregnancy is associated with increased risk for severe illness due to the SARS-CoV-2 infection. Pregnant patients with COVID-19 are more likely to need intensive care unit admission, invasive ventilation and there is an increased risk for preterm birth ^18– 20^. The ongoing discussion whether or not asthma should be considered a risk factor for severe COVID-19 is not yet closed ^21^. Nevertheless, despite critical gaps in our current knowledge, pregnant patients with asthma and COVID-19 should be managed by a multidisciplinary approach to optimize maternal and neonatal outcomes ^22^. Moreover, offering vaccination with one of the two messenger RNA (mRNA) vaccines to all pregnant and breastfeeding women is recently recommended ^23^.

A limitation of these case reports is their relatively short duration of follow-up and the fact that describing these cases cannot lead to conclusions regarding causality. Furthermore, no previous data and diagnostic asthma tests were available, so we cannot fully exclude a diagnosis of asthma prior to these pregnancies. However, based on the extensive medical history of both patients this seems unlikely.

In summary, new onset asthma should be part of the differential diagnosis of pregnant patients with respiratory symptoms, especially when mycoplasma infection is found. Uncontrolled asthma during pregnancy triggered by infection is associated with adverse pregnancy outcomes in mother and child. This should alert clinicians to seriously evaluate dyspnoea during pregnancy by physical examination, repetitive spirometry and inflammatory biomarkers such as FeNO and blood eosinophils.

More studies are needed to determine the relation between respiratory tract infections and new onset asthma during pregnancy.

## Data availability

All data underlying the results are available as part of the article and no additional source data are required.

## Consent

Written informed consent for publication of the patients’ clinical details was obtained from both patients.

This research was conducted ethically in accordance with the World Medical Association Declaration of Helsinki.
