# Supplementary material for: New onset asthma during pregnancy: two case reports
Source: F1000Res. 2023 Mar 1;10:1120. Originally published 2021 Nov 8. [Version 2] doi: 10.12688/f1000research.73188.2 (PMC10191198; doi:10.12688/f1000research.73188.2)
Supplement: Peer review history

# Peer review: Reviewer response for version 2

**DOI:** 10.5256/f1000research.144449.r165303

**Published:** 2023-04-04

**Authors:** Annika W. M. Goorsenberg

**Affiliations:** 1 Department of Respiratory Medicine, Amsterdam University Medical Center, University of Amsterdam, Amsterdam, The Netherlands

This case report study extensively describes the presentation and diagnostic process of two pregnant dyspnoic patients. This is an important topic in which large knowledge gaps are still present. Although both cases are quite different (other trimesters, readmission in the second case), the authors are certain both patients have newly onset asthma during pregnancy. 

 I agree with reviewer 1 that the differential diagnosis with airway hyperresponsiveness after (viral) infections should also be taken into account. Especially as follow-up is short as the authors conclude themselves. The adjustments made regarding this topic, adding the important limitations of these cases in the discussion, are sufficient in my opinion.

Are enough details provided of any physical examination and diagnostic tests, treatment given and outcomes?

Yes

Is the background of the cases’ history and progression described in sufficient detail?

Yes

Is sufficient discussion included of the importance of the findings and their relevance to future understanding of disease processes, diagnosis or treatment?

Yes

Is the conclusion balanced and justified on the basis of the findings?

Yes

Reviewer Expertise:

Severe asthma, endoscopic treatments for pulmonary diseases, endobronchial imaging

I confirm that I have read this submission and believe that I have an appropriate level of expertise to confirm that it is of an acceptable scientific standard.

# Peer review: Reviewer response for version 2

**DOI:** 10.5256/f1000research.144449.r165088

**Published:** 2023-03-15

**Authors:** Dominick Shaw

**Affiliations:** 1 NIHR Nottingham Biomedical Research Centre, Division of Respiratory Medicine, School of Medicine, University of Nottingham, Nottingham, UK

They have addressed my comments appropriately and amended the discussion paragraph suitably. I’m happy to approve the submission.

Are enough details provided of any physical examination and diagnostic tests, treatment given and outcomes?

Yes

Is the background of the cases’ history and progression described in sufficient detail?

Yes

Is sufficient discussion included of the importance of the findings and their relevance to future understanding of disease processes, diagnosis or treatment?

Yes

Is the conclusion balanced and justified on the basis of the findings?

Partly

Reviewer Expertise:

Asthma, digital health

I confirm that I have read this submission and believe that I have an appropriate level of expertise to confirm that it is of an acceptable scientific standard.

# Peer review: Reviewer response for version 1

**DOI:** 10.5256/f1000research.76824.r99613

**Published:** 2021-11-24

**Authors:** Dominick Shaw

**Affiliations:** 1 NIHR Nottingham Biomedical Research Centre, Division of Respiratory Medicine, School of Medicine, University of Nottingham, Nottingham, UK

This paper describes two interesting case reports where pregnant women develop features consistent with asthma. Features described include wheeze, variable FEV1 and breathlessness. Certainly, both presentations may be caused by new onset asthma but it's difficult to exclude the contribution of respiratory infection as both patients had evidence of respiratory pathogens.

 Airway infection, particularly with viruses, is well known to cause airway hyperresponsiveness (AHR). AHR can occur for several months after initial infection and is associated with similar symptoms to asthma. Some authors argue that airway hyperresponsiveness alone is enough to make a diagnosis of asthma, but other purists may argue that asthma is a combination of airway inflammation and inflammation. Either way it would be interesting to know whether symptoms persist following treatment, or resolution, of the underlying infection. Other factors pointing towards asthma would include an elevated blood eosinophil count and degree of reversibility in FEV1.

 Some factors make a diagnosis of asthma less likely-both cases had normal FEV1/FVC ratios (which reflect the effect on lung function of a gravid uterus); initial response to high dose oral Prednisolone was poor in the first case and lastly asthma symptoms seemed to improve after delivery.

 Both these cases nicely demonstrate the difficulty of making an asthma diagnosis and the physiological effects of a gravid uterus on both asthma and normal respiratory physiology.

Are enough details provided of any physical examination and diagnostic tests, treatment given and outcomes?

Yes

Is the background of the cases’ history and progression described in sufficient detail?

Yes

Is sufficient discussion included of the importance of the findings and their relevance to future understanding of disease processes, diagnosis or treatment?

Yes

Is the conclusion balanced and justified on the basis of the findings?

Partly

Reviewer Expertise:

Asthma

I confirm that I have read this submission and believe that I have an appropriate level of expertise to confirm that it is of an acceptable scientific standard, however I have significant reservations, as outlined above.

# Response

**Published:** 2022-01-09

**Authors:** Paula Schriek

**Affiliations:** Haga Teaching Hospital, The Netherlands

Dear Dominick Shaw,

 We would like to thank the reviewer for her time and critical evaluation of our manuscript entitled “New onset asthma during pregnancy: two case reports”. We also appreciate the time and effort you have dedicated to providing insightful feedback on ways to strengthen our paper. We have incorporated changes that reflect the detailed suggestions you have provided. To facilitate your review of our revisions, the following is a point-by-point response to the questions and comments delivered in your e-mail dated 2021-11-24.

 On behalf of all authors,

 Sincerely,

 Paula Schriek

 HAGA Teaching Hospital

 Department of Respiratory medicine          

 Els Borst-Eilersplein 275

 2545 AA The Hague, The Netherlands

 Phone no.: (0031) 702104392, (0031) 639592319

 Fax no.: (0031) 702102150

 E-mail: p.schriek@hagaziekenhuis.nl

**Reviewer 1**

 Airway infection, particularly with viruses, is well known to cause airway hyperresponsiveness (AHR). AHR can occur for several months after initial infection and is associated with similar symptoms to asthma. Some authors argue that airway hyperresponsiveness alone is enough to make a diagnosis of asthma, but other purists may argue that asthma is a combination of airway inflammation and inflammation.

 Either way it would be interesting to know whether symptoms persist following treatment, or resolution, of the underlying infection. Other factors pointing towards asthma would include an elevated blood eosinophil count and degree of reversibility in FEV1.

*We fully agree the importance of a clear confirmation of the diagnosis of asthma. Especially as we are well aware of the under- and over-diagnosis of asthma worldwide. (Aaron SD et al. Underdiagnosis and Overdiagnosis of Asthma. Am J Respir Crit Care Med. 2018 Oct 15;198(8):1012-1020)*

*Based on our clinical experience with patients with asthma and pregnancy (working in a centre of excellence for severe asthma and our asthma-pregnancy outpatient clinic with a monthly multidisciplinary meeting for pregnant asthmatic patients), we believe these 2 patients have asthma and not “solely” post viral AHR. We agree it would be more convincing to support this by additional objective parameters.*

*Unfortunately we were not able to perform these additional diagnostic tests or perform a longer follow-up in both patients.  However we think your consideration of AHR is of value to add to our considerations in the paper. We added this to the discussion section in the manuscript.*

 Some factors make a diagnosis of asthma less likely-both cases had normal FEV1/FVC ratios (which reflect the effect on lung function of a gravid uterus); initial response to high dose oral Prednisolone was poor in the first case and lastly asthma symptoms seemed to improve after delivery.

*We are not sure if we understood this comment as FEV1/FVC ratio is unaffected during the course of pregnancy (references mentioned below).*

*In the first case  spirometry demonstrates significant reversibility, strongly elevated FeNO and elevated blood eosinophils levels which is compatible with asthma.*

*In the second case spirometry demonstrates variability of FEV1, FEV1/FVC ratio (one time reduced with Z-score of -2.53), significant reversibility, strongly elevated FENO and blood eosinophils of 0.36×109/L under ICS, which is also compatible with asthma.*

*References:*

- 1. Weinberger SE, Weiss ST, Cohen WR, et al. . Pregnancy and the lung. *Am Rev Respir Dis* 1980; 121: 559–581
-  2. Jensen D, Webb KA, Davies GA, et al. . Mechanical ventilatory constraints during incremental cycle exercise in human pregnancy: implications for respiratory sensation. *J Physiol* 2008; 586: 4735–4750.
- 3. Jensen D, Duffin J, Lam YM, et al. . Physiological mechanisms of hyperventilation during human pregnancy. *Respir Physiol Neurobiol* 2008; 161: 76–86
- 4. McAuliffe F, Kametas N, Costello J, et al. . Respiratory function in singleton and twin pregnancy. *BJOG* 2002; 109: 765–769
- 5. Grindheim G, Toska K, Estensen ME, et al. . Changes in pulmonary function during pregnancy: a longitudinal cohort study. *BJOG* 2012; 119: 94–101

Revision of Discussion (paragraph 7, line 3-7)

 A limitation of these case reports is their relatively short duration of follow-up and the fact that describing these cases cannot lead to conclusions regarding causality. Furthermore, airway infection, particularly with viruses (like RSV or rhinovirus), may also cause airway hyperresponsiveness (AHR), although in the cases described in this report other biomarkers as FeNO and blood eosinophils are more compatible with the diagnosis of asthma(24). The exact role of viral respiratory tract infections  in the onset or progression of asthma remains a subject of debate and in this setting should be considered on a case by case basis . Lastly, no previous data and diagnostic asthma tests were available, so we cannot fully exclude a diagnosis of asthma prior to these pregnancies. However, based on the extensive medical history of both patients this seems unlikely.

 24.       Empey DW, Laitinen LA, Jacobs L, Gold WM, Nadel JA. Mechanisms of bronchial hyperreactivity in normal subjects after upper respiratory tract infection. Am Rev Respir Dis. 1976;113(2):131-9.
